# Cell Source-Dependent In Vitro Chondrogenic Differentiation Potential of Mesenchymal Stem Cell Established from Bone Marrow and Synovial Fluid of *Camelus dromedarius*

**DOI:** 10.3390/ani11071918

**Published:** 2021-06-28

**Authors:** Young-Bum Son, Yeon Ik Jeong, Yeon Woo Jeong, Mohammad Shamim Hossein, Per Olof Olsson, Alex Tinson, Kuhad Kuldip Singh, Sang-Yun Lee, Woo Suk Hwang

**Affiliations:** 1 UAE Biotech Research Center, Abu Dhabi 30310, United Arab Emirates; ybs@adbrf.org (Y.-B.S.); youniks@adbrf.org (Y.I.J.); doctorj1@adbrf.org (Y.W.J.); shamim0976@gmail.com (M.S.H.); omolson@gmail.com (P.O.O.); 2 Hilli E.T. Cloning and Surgical Centre Presidential Camels and Camel Racing Affairs, Al-Ain 17292, United Arab Emirates; heffoundation@hotmail.com (A.T.); kskuhad@hotmail.com (K.K.S.); 3 Department of Theriogenology and Biotechnology, College of Veterinary Medicine and Research Institute of Life Science, Gyeongsang National University, Jinju 52828, Korea; sy_lee@gnu.ac.kr

**Keywords:** mesenchymal stem cells, *Camelus dromedarius*, bone marrow, synovial fluid, chondrocyte differentiation

## Abstract

**Simple Summary:**

This is the first study to demonstrate the establishment and subsequent analysis of attributes, including the chondrogenic capacity of mesenchymal stem cells (MSCs) from bone marrow (BM) and synovial fluid (SF) from the same donor *Camelus dromedarius*. MSCs of SF origin were notably more efficient in their chondrogenic capacity and represent a potential source for camel regenerative medicine addressing chondrocyte-related problems.

**Abstract:**

Mesenchymal stem cells (MSCs) are promising multipotent cells with applications for cartilage tissue regeneration in stem cell-based therapies. In cartilage regeneration, both bone marrow (BM-MSCs) and synovial fluid (SF-MSCs) are valuable sources. However, the cellular characteristics and chondrocyte differentiation potential were not reported in either of the camel stem cells. The in vitro chondrocyte differentiation competence of MSCs, from (BM and SF) sources of the same *Camelus dromedaries* (camel) donor, was determined. Both MSCs were evaluated on pluripotent markers and proliferation capacity. After passage three, both MSCs showed fibroblast-like morphology. The proliferation capacity was significantly increased in SF-MSCs compared to BM-MSCs. Furthermore, SF-MSCs showed an enhanced expression of transcription factors than BM-MSCs. SF-MSCs exhibited lower differentiation potential toward adipocytes than BM-MSCs. However, the osteoblast differentiation potential was similar in MSCs from both sources. Chondrogenic pellets obtained from SF-MSCs revealed higher levels of chondrocyte-specific markers than those from BM-MSCs. Additionally, glycosaminoglycan (GAG) content was elevated in SF-MSCs related to BM-MSCs. This is, to our knowledge, the first study to establish BM-MSCs and SF-MSCs from the same donor and to demonstrate in vitro differentiation potential into chondrocytes in camels.

## 1. Introduction

Cartilage damage to joint surfaces can result in osteoarthritis (OA), a condition where the bone under the articular cartilage is exposed and the synovial membrane around the joint is inflamed [1]. When cartilage is damaged due to inflammation or trauma, it cannot buffer between the bone, causing severe pain and deformation of the articular cartilage. OA is a chronic musculoskeletal disorder frequently occurring in racing horses and camels that adversely affects their future racing careers [2,3].

Since cartilage has no distribution of blood vessels and nerves, it is difficult to regenerate once damaged [4]. To treat damaged cartilage tissue, drugs (steroids and painkiller), chondroprotective agents (hyaluronic acid and glucosamine), and surgical approaches are used [5]. However, limited effects of non-specific alleviation of pain and inflammatory reactions are expected in drug treatment and chondroprotective agents only play a role in supplying nutrients to chondrocytes [1,6]. Microfracture and autologous chondrocyte implantation (ACI) have been performed as clinical surgical methods based on cell therapy [7]. Microfracture is a method of regenerating cartilage with blood clots containing mesenchymal stem cells (MSCs) by puncture or abrasion of damaged subchondral bone and has the disadvantages of regenerating into fibrocartilage rather than hyaline cartilage [8]. ACI, which is commonly used, induces the regeneration of cartilage tissue by transplanting autologous cartilage cell culture in vitro [8]. However, this method has specific problems, including the formation of cartilage defects in macroscopically normal-looking tissue donor sites. Furthermore, the phenotype can change due to de-differentiation during in vitro culture since the transplanted cells are already differentiated cells [9,10]. Therefore, therapeutic approaches have emerged for cartilage regeneration using stem cells. 

MSCs are a potent resource for therapeutic application with a high stemness ability, anti-inflammatory, and immunosuppressive effects [11,12]. MSCs also possess multi-lineage differentiation capacity including chondrocytes [13]. Bone marrow stem cells (BM-MSCs), which were the first to be discovered, can mainly be collected from the iliac crest of the pelvis, and have been reported for a long time. However, several disadvantages have been reported, such as the limited amount of harvest and the complicated collection process [14]. Furthermore, compared to other sources, low proliferative capacity and donor-age-dependent characteristics have been reported in BM-MSCs [15]. Considering the shortcomings of BM-MSCs, studies have been performed on alternative MSCs sources.

Synovial fluid-derived MSCs (SF-MSCs) can be collected by the process of arthrocentesis and possess superior chondrogenic capacity [16]. SF-MSCs are the most similar source to articular cartilage, have a high expression of CD44 (hyaluronan receptors) and uridine disphosphoglucose dehydrogenase (UDPGD), and are required for hyaluronan synthesis [17]. Accordingly, several studies have reported on cartilage regeneration using SF-MSCs in various mammalians [18,19,20]. Human SF-MSCs derived from OA patients were transplanted with a collagen sponge into the joint of the anterior cruciate ligament transection of rats and cartilage defects were recovered [18]. SF-MSCs derived from equine sources have also been shown to have an analogous beneficial effect compared to BM-MSCs after implantation into articular cartilage defects in rat femurs [19]. An interesting study reported that after the transplantation of porcine SF-MSCs into collagen-induced arthritis mouse (CIA mouse) by intraperitoneal injection, cartilage damage in the joint was decreased, and inflammation was also reduced [20]. Nevertheless, a comparison of these two cell types is lacking in *Camelus dromedarius* (camel).

In this study, we established a distinctive population of BM-MSCs and SF-MSCs derived from the same donor. Additionally, to evaluate chondrogenic potential, both camel MSCs were differentiated into chondrocytes under specific induction conditions. 

## 2. Materials and Methods

### 2.1. Chemicals and Media

Unless otherwise stated, all reagents were obtained from Sigma (St. Louis, MO, USA).

### 2.2. Collection and Culture of Bone Marrow and Synovial Fluid Derived Mesenchymal Stem Cells

Institutional Animal Care and Use Committee (IACUC) guidelines were followed and all experiments were approved by the Management of Scientific Centers and Presidential Camels (Accession No: PC4.1.5). Four female camels aged between 5 and 7 years were used to establish and compare the various MSCs. After placing the camels in a seated position, they were sedated with ketamine hydrochloride (Ilium, Hlendenning, Australia; 0.25 mg/kg body weight) and xylazine (Ceva, Libourne, France; 0.25 mg/kg body weight) through intravenous injection using an 18-gauge needle [13]. The 2% lidocaine (Jeil, Daegu, Korea) was infiltrated subcutaneously on the iliac crest (2 cm × 2 cm). Approximately 5 mL of BM was extracted using the 11-gauge, 15-cm-long biopsy needle (Argon Medical Devices, Frisco, TX, USA). A heparin-coated 10 mL syringe, was used for BM extraction and subsequently added to the same quantity of Dulbecco’s phosphate-buffered saline (DPBS, Welgene, Gyeongsan, Korea). Centrifugation was performed at 400× *g* for 40 min, layered onto Ficoll-Paque (Amersham Biosciences, Uppsala, Sweden) without mixing. Mononuclear cells (MNCs) were harvested from the interface buffy layers and washed with DPBS. 

We collected the SF using syringe aspiration as previously reported with minor modifications [21]. In brief, a hypodermic 18-gauge needle was used to gently aspirate 5 mL of SF from the femorotibial joint with a 10 mL syringe. A nylon filter with a pore size of 40 µm (Falcon, Franklin, NJ, USA) was used to separate debris before cell isolation through centrifugation at 400 g for 10 min. Cells were cultured with DMEM, high-glucose, 10% FBS, and 1% of each nonessential amino acids, antibiotic-antimycotic, and 0.1% β-mercaptoethanol (Thermo Fisher Scientific, Waltham, MA, USA). Incubator conditions were 38 °C with high humidity, 5% O_2_, and 5% CO_2_. Culture media were exchanged every second day until 80% cellular confluence was obtained. Subculture and cryopreservation were performed using a 0.25% trypsin EDTA solution (Gibco, Paisely, UK) and a cryopreservation solution using the same DMEM used for cell culture with 20% FBS and 10% dimethyl sulfoxide (DMSO).

### 2.3. Proliferation and Cell Cycle Assay

Cell cycle assay, including population doubling time (PDT) was conducted as previously reported with minor modifications [22]. Six-well plates (Nunc, Rochester, NY, USA) were used for seeding MSCs and cultured in an incubator as described above. A hematocytometer was used corresponding to passage intervals, at 72-h, to determine cell counts. PDT was established as follows: PDT = log2 × T/(logNC − logNI): T is culture time, logNC is the cell number at the time, and logNI is the initial cell count.

Cells were fixed at passage 3 using 70% ethanol for 1 h. A 10 μg/mL propidium iodide solution was used with 1 × 10^6^ fixed cells for 15 min, including RNase A (100 μg/mL). Analysis and categorization into cell stage was performed via flow cytometry (BD FACSVerseTM, BD Biosciences, San Jose, CA, USA). All experiments were conducted in 4 independent replicates.

### 2.4. In Vitro Differentiation into Trilineage and Cytochemical Staining

Trilineage (osteoblast, adipocyte, and chondrocyte) differentiation was conducted using BM-MSCs and SF-MSCs as previously described [13]. For osteogenic differentiation, cells were cultured for a total of 21 days in a DMEM media supplemented with 10% FBS 10 nM dexamethasone, 50 μg/mL ascorbic acid, and 10 mM sodium beta-glycerophosphate. After differentiation, mineralization and calcium deposition were affirmed using Alizarin red S and von Kossa staining. Adipogenic differentiation of cultured MSCs was conducted in DMEM supplemented with10% FBS, 100 µM indomethacin, 10 µM insulin, and 1 µM dexamethasone. Differentiation into chondrocytes was accomplished utilizing a modified pellet culture method. Pellets were made in 15 mL centrifuge tubes with 1 mL cellular suspension of passage 3 cells (1 × 10^6^) in STEMPRO chondrogenesis differentiation media with 10% supplement in a 15 mL tube. Centrifugation was performed at 450× *g* for 5 min and the resultant pellets were cultured for 3 weeks. Afterward, culture pellets were embedded in paraffin and sectioned on glass slides following dehydration. A 1% Alcian blue solubilized in 3% acetic acid was used for staining for a period of 10 min with proteoglycan deposition confirmed with a 1 min counterstaining using a 0.1% solution of nuclear fast. 

### 2.5. Real-Time Quantitative Polymerase Chain Reaction (RT-qPCR) Analysis

The expressions of pluripotent markers, cluster of differentiation (CD) markers, and lineage-specific genes were analyzed. An easy-spin Total RNA Extraction Kit (Intron, Seongnam, Korea) was used for total RNA extraction. Nucleotide quantification was performed with a nanodrop 1000 spectrophotometer (Thermo Fisher Scientific, Waltham, MA, USA). Complementary DNA was synthesized with 2 μg purified RNA and reverse transcription with a HisenScript RT PreMix kit (Intron, Seongnam, Korea); synthesis was performed over 50 min at 42 °C with 10 μM OligodT primers. A Rotor-Gene Q cycler (Qiagen, Hilden, Germany) was used for RT qPCR and RealMODTM Green AP 5× qPCR mix (Intron, Seongnam, Korea) containing 200 nM primers (Table 1). Amplification was performed by denaturation at 95 °C for 60 s and subsequently 50 cycles of 95 for 10 min, 60 °C for 6 s, and 72 °C for 4 s. Expression was normalized to mRNA levels of glyceraldehyde-3-phosphate dehydrogenase (GAPDH). PCR products were analyzed by electrophoresis in 1% agarose gels and ethidium bromide staining. Amplified products were visualized under UV light. All experiments were conducted in 4 independent replicates.

### 2.6. Glycosaminoglycan (GAG) Contents

The differentiated chondrocyte pellets from SF-MSCs and BM-MSCs were stored at −20 °C following washing prior to glycosaminoglycan (GAG) content assay. Cell pellets were digested with 0.5 mg/mL papain solution and proteoglycan content was determined using the dimethylmethylene blue (DMMB) spectrophotometric assay. Chondroitin-4 sulfate (Sigma-Aldrich, St. Louis, MO, USA) was used to establish a standard curve. The optical density of 525 nm was used on a microplate reader (VersaMax, Molecular Devices, Sunnyvale, CA, USA). A DNA quantification kit (Abcam, Cambridge, UK) was used to determine the DNA content. All experiments were conducted in 4 independent replicates.

### 2.7. Statistical Analysis

Data analysis was performed with SPSS version 23 (IBM) for independent T-tests and one-way analysis of variance (ANOVA), and intergroup differences were identified with Tukey’s test. Data are represented as the mean ± standard deviation (SD), and a *p* value < 0.05 is considered significant.

## 3. Results

### 3.1. Establishment of MSCs Derived from Bone Marrow and Synovial Fluid

**Figure 1:**
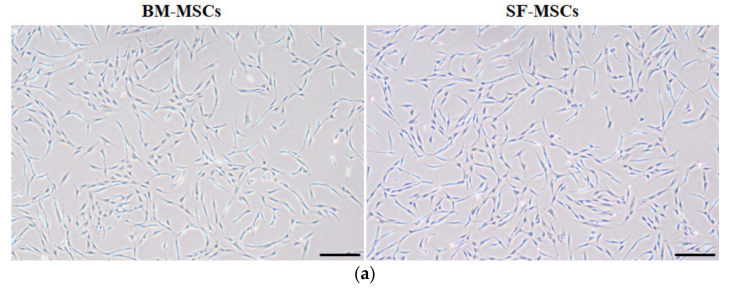

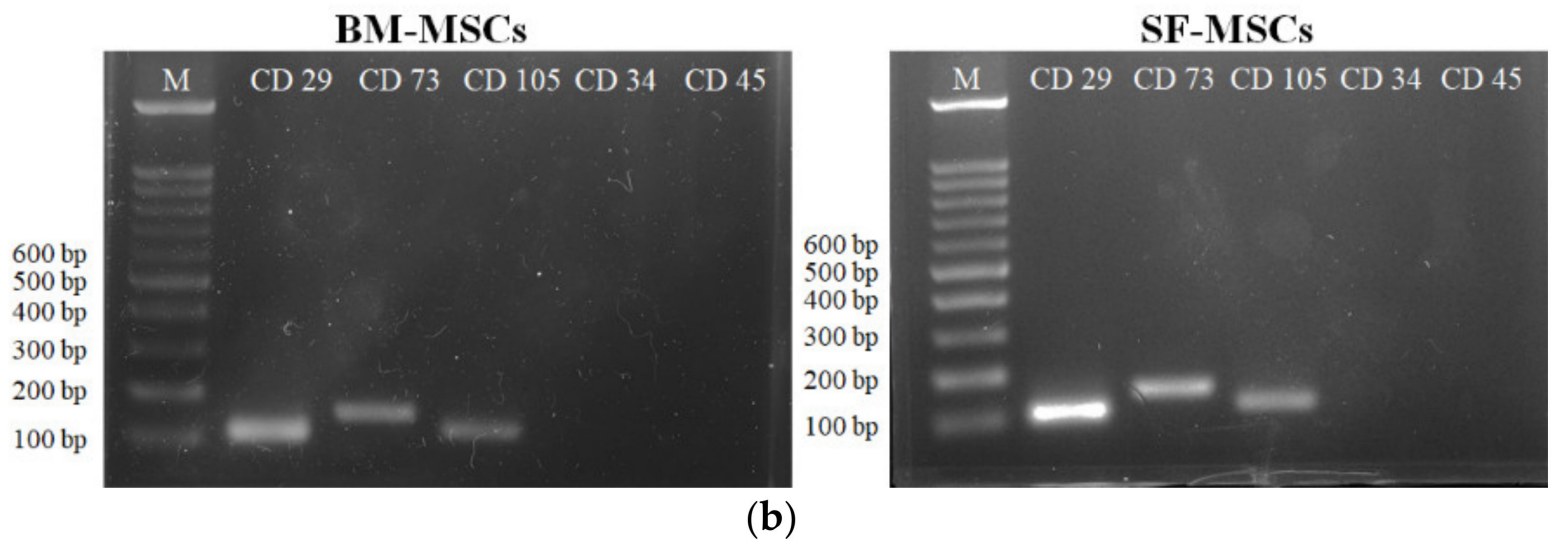
Establishment of BM-MSCs and SF-MSCs. (**a**) Cellular morphology of BM-MSCs and SF-MSCs from camel. Both MSCs showed a spindle-like morphology at passage 3. Scale bar = 100 µm. (**b**) Agarose gel electrophoresis of PCR products. Both BM-MSCs and SF-MSCs were positive for the mesenchymal stem cell markers (CD29, CD72, and CD105) and were negative for the hematopoietic stem cell markers (CD34 and CD45). Lane M: 100 bp DNA ladder.

**Figure 2:**
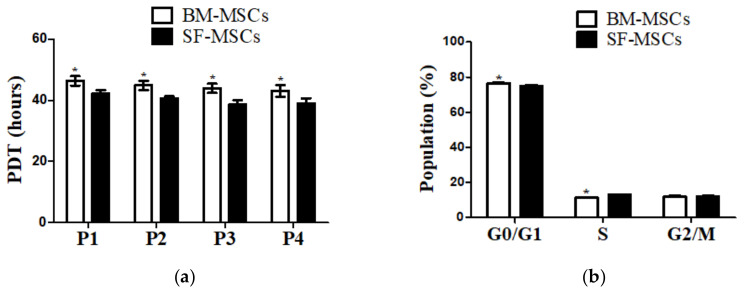
Cell cycle and proliferation capacity of bone marrow (BM-MSCs) and synovial fluid (SF-MSCs) from the camel. (**a**) The proliferation capacity was evaluated by population doubling time (PDT) assay. SF-MSCs showed increased proliferation capacity compared to BM-MSCs during the passage. (**b**) Cell cycle analysis indicated that cell arrest and DNA replication were significantly increased in SF-MSCs compared to BM-MSCs. Bar graphs illustrate mean values +/− SD (*n* = 4). * denote significant (*p* < 0.05) differences (P1, P2, P3, and P4: passage 1, 2, 3, and 4; G0/G1: G0/G1 phase; S: S phase; G2/M: G2/M phase).

**Figure 3:**
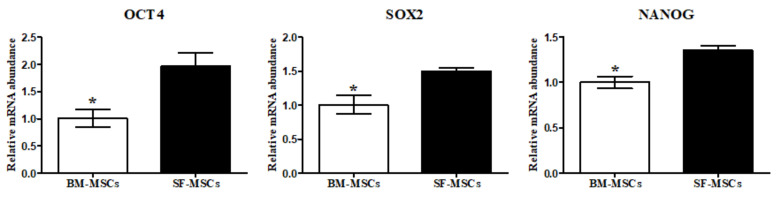
Stem cell transcription factor expression of BM- and SF-MSCs. SF-MSCs when related to BM-MSCs exhibited increased expression for all three transcription factors. * denote significant differences (*p* < 0.05). Bar graphs illustrate mean values +/− SD (*n* = 4).

We isolated and cultured MSCs derived from BM and SF from the same donors. MSCs from both tissue sources exhibited spindle-like morphology and homogenous attachments to culture surfaces were confirmed after passage 3 (Figure 1a). The results of RT-PCR showed that BM-MSCs and SF-MSCs were positive for the mesenchymal stem cell markers (CD 29, CD73, and CD105) and were negative for the hematopoietic stem cell markers (CD34 and CD45) (Figure 1b). To confirm the proliferation capacity of cells, we analyzed PDT. SF-MSCs showed higher proliferation potential than BM-MSCs (Figure 2a). Cell cycle analysis was used as a measure of cellular viability, at the third passage. We observed a significantly increased proportion of S phase and diminished G0/G1 phase in SF- compared to BM-derived MSCs (Figure 2b). Compared with the BM-MSCs, the transcription factors, NANOG, SOX2, and OCT4, showed significantly increased expression in the SF-derived cultures (Figure 3). 

### 3.2. In Vitro Osteogenic and Adipogenic Lineage Differentiation Potential of MSCs

**Figure 4:**
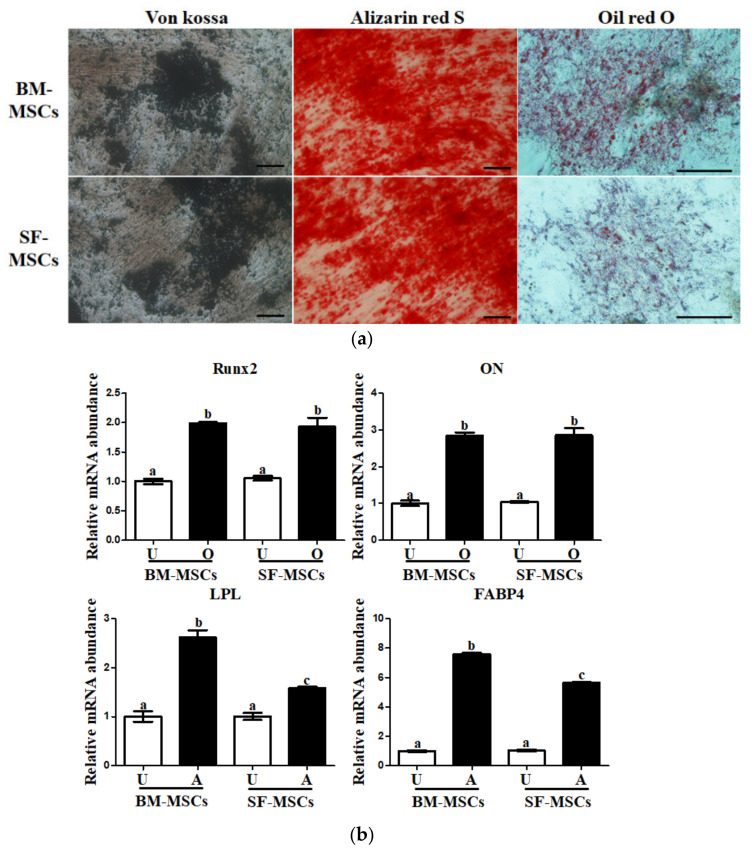
In vitro differentiation into osteoblast and adipocyte from BM- and SF- derived MSCs. (**a**) The osteoblast differentiation of both MSCs was confirmed by staining of calcium deposition and mineralization with von Kossa and Alizarin red S (Scale bar = 100 µm). (**b**) Osteoblast (Runx2 and ON) and adipocyte (LPL and FABP4) related gene expression in induced cells. All adipocyte-related genes were significantly increased in differentiated cells from BM-MSCs compared to those from SF-MSCs. However, there was no difference in the expression of osteoblast-related genes between the BM- and SF-MSCs. Graphs represent mean data ± SD from independent experiments (*n* = 4). Different superscripts (a, b, and c) denote significant differences (*p* < 0.05) among the undifferentiated control and differentiated cells using BM-MSCs and SF-MSCs (white bar: undifferentiated cells; black bar: differentiated cells; U: undifferentiated cells; O: osteogenic differentiated cells; A: adipogenic differentiated cells).

To evaluate the influence of the source of MSCs on the differentiation capacity, BM-MSCs and SF-MSCs were differentiated into osteoblasts and adipocytes. The calcified extracellular matrix formation which is indicative of osteoblast differentiation was confirmed using Alizarin red S and von Kossa staining, and the presence of intracellular lipid droplets vacuoles, which is indicative of adipocyte differentiation, was confirmed using Oil red O staining. All differentiation processes were confirmed using both BM-MSCs and SF-MSCs (Figure 4a). 

The expression of osteogenesis- and adipogenesis-related genes were analyzed pre- and post- differentiation. Gene expression associated with osteogenesis and adipogenesis was investigated pre- and post-differentiation. The expression of osteoblast-related expression, i.e., runt-related transcription factor 2 (Runx2), osteocalcin (ON), in all measured MSCs, significantly (*p* < 0.05) increased after differentiation (Figure 4b). However, there was no significant (*p* < 0.05) difference in the expression of Runx2 and ON in the differentiated osteoblasts from the two groups (Figure 4b). A significant increase was observed in adipocyte differentiation, lipoprotein lipase (LPL), and the fatty acid-binding protein 4 (FABP4) in the differentiated adipocytes derived from SF-MSCs compared to BM-MSCs (Figure 4b).

### 3.3. In Vitro Chondrogenic Differentiation Capacity of MSCs

**Figure 5:**
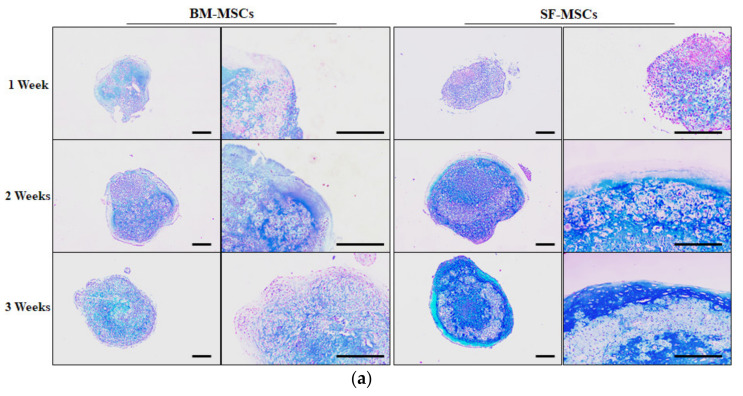

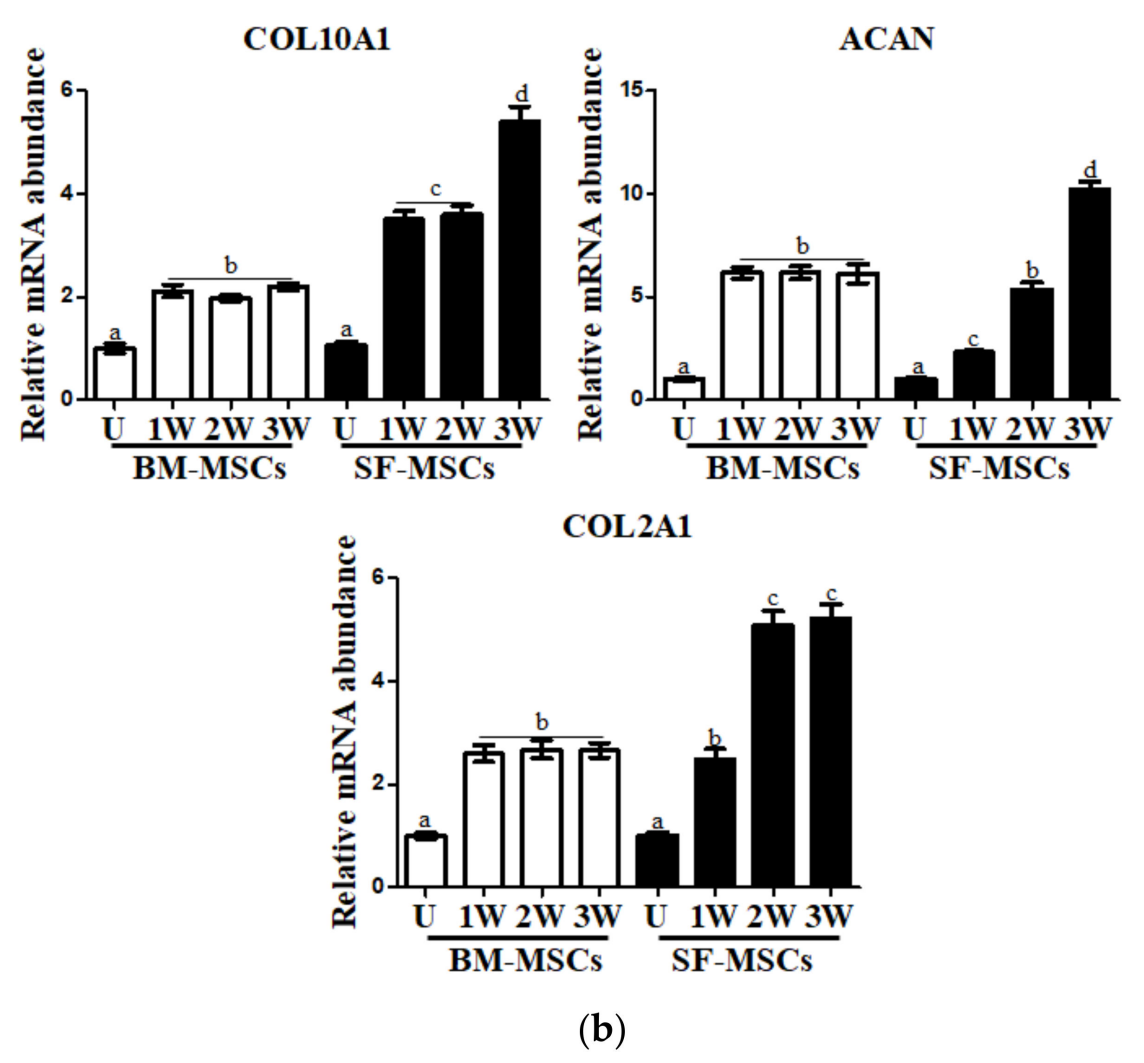
In vitro chondrocyte differentiation capacity by time of BM- and SF-MSCs. Chondrogenesis was evaluated by cytochemical staining and RT-qPCR analysis. (**a**) In vitro chondrocyte differentiation was demonstrated by Alcian blue staining in induced pellets one, two, and three weeks after induction. Scale bar = 500 µm. (**b**) Genes (COL10A1, ACAN, and COL2A1) expressed in chondrocytes, in induced BM- and SF-MSCs one, two, and three weeks after induction. At three weeks following chondrogenesis, all chondrocyte-specific markers were significantly increased in chondrocyte pellets in SF- versus BM-MSCs. Values displayed as mean and ± SD, independent experiments (*n* = 4). Different superscripts (a, b, c, and d) denote significant differences (*p* < 0.05) among undifferentiated cells, and 1, 2, and 3 weeks of differentiated cells using BM-MSCs and SF-MSCs (white bar: undifferentiated cells; black bar: differentiated cells U: undifferentiated cells; 1W: one week after chondrocyte induction; 2W: two weeks after chondrocyte induction; 3W: three weeks after chondrocyte induction).

**Figure 6:**
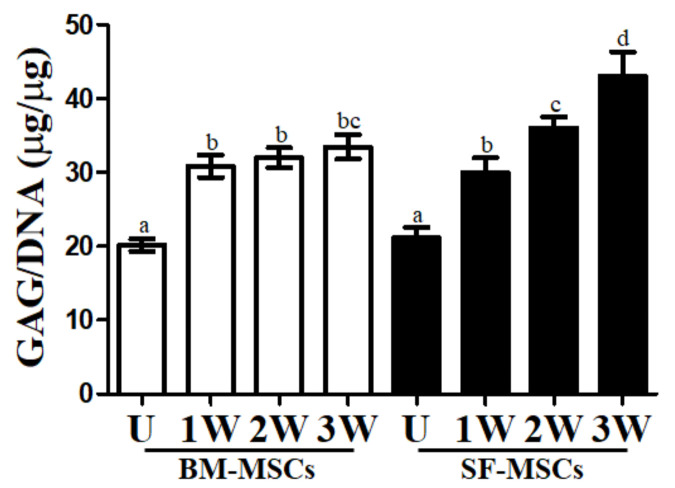
The values of glycosaminoglycan (GAG) content of BM- and SF-MSCs was evaluated one, two, and three weeks after induction. The GAG contents were significantly higher in groups following differentiation in SF compared to BM-MSCs after three weeks of differentiation. Values displayed are mean ± SD independent experiments (*n* = 4). Different superscripts (a, b, c, and d) denote significant differences (*p* < 0.05) among undifferentiated cells, and 1, 2, and 3 weeks of differentiated cells using BM-MSCs and SF-MSCs (white bar: undifferentiated cells; black bar: differentiated cells U: undifferentiated cells; 1W: one week after chondrocyte induction; 2W: two weeks after chondrocyte induction; 3W: three weeks after chondrocyte induction).

Differentiation of BM- and SF-MSCs into chondrocytes succeeded using the pellet culture method. The accumulation of proteoglycan was observed both in pellets from BM- and SF-MSCs after 1 week by Alcian blue staining (Figure 5a). Following the differentiation process, these cells grew and the multi-layered structure was confirmed in the chondrogenic pellet. Some portion of the pellet showed hypertrophic chondrocyte formation after 2 to 3 weeks on chondrocyte differentiation using SF-MSCs (Figure 5a). The expression levels of type X collagen gene (COL10A1), aggrecan (ACAN), and the alpha 1 chain of collagen type II (COL2A1) were investigated following three weeks of the chondrogenesis procedure (Figure 5b). In BM-MSCs, the levels of chondrocyte-specific gene expressions were significantly higher in differentiated chondrocytes at 1 to 3 weeks compared to undifferentiated cells (Figure 5b). There was a more significant expression in all chondrocyte-specific markers analyzed in differentiated cells following the chondrogenesis protocol in SF- compared to BM-MSCs. The levels of ACAN gradually increased as the chondrogenesis from SF-MSCs. In the process of differentiation of BM-MSCs into chondrocytes, COL10A1, ACAN, and COL2A1 expression were not significantly different. The deposition of glycosaminoglycan (GAG) was increased in chondrogenic pellets related to undifferentiated pellets (Figure 6). Furthermore, after two weeks of differentiation into chondrocytes, SF-MSCs showed increased GAG deposition compared to BM-MSCs (Figure 6).

## 4. Discussion

Articular cartilage is fibrillar connective tissue distributed throughout the musculoskeletal system. It connects two bones and acts as a buffer against mechanical stress [4]. Chondrocytes differentiate from mesenchymal cells during development to form cartilage tissue. Unlike other tissue, cartilage consists of only one type of chondrocyte, and the formed cartilage tissue remains as a permanent cartilage tissue as with articular cartilage [4]. Cartilage damage caused by trauma has frequently been reported in racing camels that perform vigorous exercise, which develops into chronic OA in the absence of appropriate treatment. There is a limit to the natural recovering of the articular cartilage once it is damaged, and many treatments are currently applicable. Generally, these disorders are treated by autologous chondrocyte implantation (ACI) [23,24]. However, the effect of ACI is limited in broad degenerative arthritis and elderly patients. Considering the limitation of ACI, an alternative therapy based on stem cells has emerged for chondrocyte regeneration. 

Ideal sources of MSCs in stem cell-based therapy for OA could have enhanced chondrogenic capacity for recovering destructed cartilage. To date, BM-MSCs and SF-MSCs therapeutic uses have predominantly been applied for the regeneration of cartilage [20,25]. No studies have been reported, on the establishment and the biological characteristics including the potential to differentiate MSCs into chondrocytes, in camels. This study aimed to evaluate biological characteristics, such as morphology; proliferation; stemness; and trilineage differentiation potency, including chondrogenesis in camel MSCs. Both BM- and SF-MSCs were successfully established from the same donors. Both types of homogenous adherent MSCs showed fibroblastic spindle-like morphology. The expression of transcription factors and the proliferation capacity were related to the self-renewal ability of MSCs [26]. OCT4 and SOX2 are crucial early transcription factors for sustaining stemness and pluripotency and are positively expressed in MSCs with NANOG. Our data showed that SF-MSCs possessed superior proliferation capacity compared to BM-MSCs as previously reported [20,27]. The levels of NANOG, SOX2, and OCT4 were significantly increased in SF compared to BM-MSCs [20]. 

Both osteoblast and adipocyte markers were expressed from BM- and SF-MSCs differentiation protocols. Expression of the lineage-specific genes differed between BM-MSCs and SF-MSCs. Oil red O, a cytochemical stain, along with adipocyte-associated gene expressions, indicated improvements in adipocyte differentiation capacity from SF-MSCs compared to BM-MSCs. These results are similar to previous reports in miniature pigs [20]. There was a significant increase in levels of genes; i.e., Runx2 and ON, considered to be specific to osteoblasts, followed differentiation protocols in MSCs. However, there was no difference in the capacity of osteogenesis between the two groups, which is in accordance with the previous study [28].

The chondrogenesis capacity of SF-MSCs is crucial for therapeutic recovery in OA. Chondrocytes possess variable phenotypes, such as de-differentiation that loses differentiated characteristics and re-differentiation [29,30]. When articular chondrocytes are cultured with a low-density monolayer, type II collagen expression decreases rapidly, and the expression of type I and III collagen, typically associated with fibroblasts, increases [31]. However, through three-dimensional culture, collagen type II expression was promoted, suggesting re-differentiation into chondrocytes [31]. Therefore, in this study, the chondrocyte differentiation capacity of both BM- and SF-MSCs through high cell density pellet culture, was investigated. A previous study was reported that chondrocyte-specific gene expression gradually increased during the chondrogenesis process of the SF-MSCs, whereas no gradual increase was observed in BM-MSCs [20]. The present study also showed that after three weeks of induction, chondrocyte-specific gene expression gradually increased during the chondrogenesis protocol in SF-MSCs compared to BM-MSCs. Chondrocyte-associated genes, i.e., COL10A1, ACAN, and COL2A1 were elevated in SF- compared to BM-MSCs. However, some hypertrophic chondrocytes were observed during the differentiation of SF-MSCs into chondrocytes.

Cartilage is a complex tissue comprising chondrocytes; extracellular matrix (ECM) proteins, including proteoglycans and GAGs, etc., [32,33]. Various GAGs exist, including chondroitin sulfate, dermatan sulfate, keratan sulfate, decorin, and fibromodulin in cartilage, and form proteoglycan by binding with core proteins [34]. Therefore, GAG synthesis is a crucial mark of chondrogenesis. The present study showed that the values of GAG content were significantly increased in differentiated chondrocytes compared to undifferentiated cells. Chondrocytes from SF-MSCs exhibited higher values of GAG content than those from BM-MSCs. Similarly, the intensity of Alcian blue stain was greater in SF compared to BM-MSCs. Although further studies will be necessary on the efficacy of cartilage regeneration in vivo and the suppression of cartilage hypertrophy, in which the synthesis of COL10A1 is prominent is needed but SF-MSCs appear to express superior chondrogenic capacity when compared to BM-MSCs. 

The present study provides the potential stem cell-based therapy of *Camelus dromedarius* utilizing MSCs with superior chondrogenesis. The pre-establishment of MSCs can support direct treatment following cartilage-related problems such as OA caused by severe trauma. This stem cell-based therapy using MSCs promotes animal welfare by realizing injuries promptly and decreasing the risks for additional injuries.

## 5. Conclusions

In this paper, we showed the differences in proliferation capacity, expression of transcription factors, and the MSCs trilineage potential of both camel BM and SF. BM-MSCs were shown to have a more reliable osteogenic differentiation capacity compared to SF-MSCs. SF-MSCs had greater proliferative potential and expressed larger amounts of transcription factors than did BM-MSCs. After the completion of the in vitro chondrocyte differentiation, hypertrophic chondrocytes were observed in parts of the SF-MSCs. Nevertheless, the expression of chondrocyte-specific markers and GAG contents indicated that SF-MSCs showed enhanced chondrocyte differentiation capacity compared to BM-MSCs. 

This study, to the best of and our knowledge, is the first to report the establishment and properties of camel BM- and SF-MSCs from the same donor to date. The observed chondrogenic ability alludes to the potential of SF-MSCs as a target cell source for future use in therapeutic cartilage regeneration.

## Figures and Tables

**Table 1:** Lists of camel primers used in RT-qPCR analysis.

Gene name (Symbol)	Primers Sequence	Product Size (bp)	Anneal. Temp (°C)
POU class 5 homeobox 1 (OCT4)	F: CGAGAGGATTTTGAGGCTGC R: GAGTACAGTGTGGTGAAGTGAG	122	60
Sex determining region Y-box 2 (SOX2)	F: CTCGCAGACCTACATGAACG R: TGGGAGGAAGAGGAAACCAC	144	60
Nanog homeobox (NANOG)	F: AGCACAGAGAAGCAGGAAGA R: CCACCGCTTACATTTCATTC	213	60
Runt-related transcription factor 2 (Runx2)	F: GACAGAAGCTTGATGACTCT R: GTAATCTGACTCTGTCCTTG	166	60
Osteocalcin (ON)	F: AGTGAGATGGTGAAGAGACT R: TAGGTTGTGCCGTAGAAG	176	60
Lipoprotein lipase (LPL)	F: GAGAGTGTTACCTACACCAA R: GCCTTTACTCTGATCTTCTC	248	60
Fatty acid-binding protein 4 (FABP4)	F: GTGACCATCAGTGTGAATG R: GCACCTCCTTCTAAAGTTAC	152	60
The type X collagen gene (COL10A1)	F: TATCCAGCTATAGGCAGTC R: TCGTAGGTGTACATTACAGG	194	60
Aggrecan (ACAN)	F: TGTGGAGGGTGTTACTGAAC R: GACTGATGACCCTTCTACCC	154	60
Collagen type II alpha 1 chain (COL2A1)	F: GTGGTGACAAAGGTGAAAAA R: AGCCTTCTCATCAAATCCTC	154	60
Glyceraldehyde 3-phosphate dehydrogenase (GAPDH)	F: GCTGAGTACGTTGTGGAGTC R: TCACGCCCATCACAAACATG	133	60
Integrin beta-1 (CD 29)	F: CTTGCGTTGCTGCTGATTTG R: TTCTTGCGTGTCCCATTTGG	105	60
5′-nucleotidase (CD 73)	F: CAACCTCAGACATGCCGATG R: GTCAAAGGTGCCTCCAAAGG	154	60
Endoglin (CD 105)	F: TCCTCCAGACCTCCAACTCT R:CCCAAATTCAGTTGGCAGCT	115	60
Cluster of differentiation 34 molecule (CD 34)	F: GGTCTTGGCCAACAGAACAG R: CAGCTTCGACGGTTCATCAG	216	60
Protein tyrosine phosphatase, receptor type, C (CD 45)	F: AACTCTTGGCATTTGGCGTT R: TTCTGCCTACACTCAAGGGG	220	60

## Data Availability

Data access can be requested on demand from the corresponding author.

## Abbreviations

MSCs: Mesenchymal stem cells; BM: Bone marrow; SF: Synovial fluid; BM-MSCs: Bone marrow-derived mesenchymal stem cells; SF-MSCs: Synovial fluid-derived mesenchymal stem cells; GAG: Glycosaminoglycan; OA: Osteoarthritis; ACI: Autologous chondrocyte implantation; UDPGD: Uridine disphosphoglucose dehydrogenase; CIA mouse: Collagen-induced arthritis mouse; Camel: Camelus dromedarius; DPBS: Dulbecco’s phosphate-buffered saline; MNCs: Mononuclear cells; DMSO: Dimethyl sulfoxide; PDT: Population doubling time; CD marker: Cluster of differentiation marker; DMMB: Dimethylmethylene; SD: Standard deviation; Runx2: Runt-related transcription factor 2; ON: Osteocalcin; LPL: Lipoprotein lipase; FABP4: Fatty acid-binding protein 4: COL10A1: Type X collagene gene; ACAN: Aggrecan; COL2A1: Alpha 1 chain of collagen type II; ECM: Extracellular matrix.
